# Monochorionic diamniotic twins of discordant external genitalia with 45,X/46,XY mosaicism

**DOI:** 10.1002/mgg3.1382

**Published:** 2020-06-25

**Authors:** Ken Takahashi, Taisuke Sato, Miyuki Nishiyama, Aiko Sasaki, Kosuke Taniguchi, Ohsuke Migita, Seiji Wada, Kenichiro Hata, Haruhiko Sago

**Affiliations:** ^1^ Center for Maternal‐Fetal‐Neonatal and Reproductive Medicine National Center for Child Health and Development Setagaya‐ku Tokyo Japan; ^2^ Department of Maternal‐Fetal Biology National Research Institute for Child Health and Development Setagaya‐ku Tokyo Japan; ^3^ Department of Obstetrics and Gynecology The Jikei University School of Medicine Minato‐ku Tokyo Japan

**Keywords:** discordant sex, monozygotic twins, mosaicism, twin pregnancy

## Abstract

**Background:**

Monozygotic twins with 45,X/46,XY mosaicism, discordant for phenotypic sex, are extremely rare.

**Methods:**

This report describes the clinical findings of a rare case of 45,X/46,XY mosaicism in monozygotic twins with different external genitalia. Single nucleotide polymorphism (SNP) array analysis, performed by collecting DNA from each umbilical cord, showed identical SNPs in the autosomal chromosomes of both fetuses.

**Results:**

Chorionic villus sampling of a 37‐year‐old primigravida carrying monozygotic twins revealed a 45,X/46,XY karyotype. Autopsy of the aborted fetuses revealed a penis and testes on one fetus and a vagina, uterus, and ovaries in the other fetus––which also had severe cystic hygroma. Cell counting using fluorescence in situ hybridization with XY probes (XY‐FISH) showed 20% and 80% abundance of 45,X cells in the internal genitalia, liver, heart, lung, adrenal gland, bone marrow, and spine of the male and female fetuses, respectively.

**Conclusion:**

These results indicated that the fetuses were genetically monozygotic twins and their different degrees of mosaicism may have resulted in different genital phenotypes.

## Graphical abstract

This report was case of 45,X/46,XY mosaicism in monozygotic twins with different external genitalia. Cell counting using fluorescence in situ hybridization with XY probes (XY‐FISH) showed 20% and 80% abundance of 45,X cells in the internal genitalia, liver, heart, lung, adrenal gland, bone marrow, and spine of the male and female fetuses, respectively.

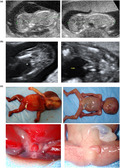

## 1 INTRODUCTION

The occurrence of the 45,X/46,XY mosaic karyotype is rare, with an incidence of approximately 0.15% at birth (Hamerton, Canning, Ray, & Smith, 1975). A broad spectrum of phenotypes, ranging from normal to the “classical” Turner syndrome, has been observed in patients with 45,X/46,XY mosaicism (Hsu, 1994). However, monozygotic twins with 45,X/46,XY mosaicism, discordant for phenotypic sex, are extremely rare. This discordance may be associated with 45,X and 46,XY mosaic cell lines (Rohrer, Gassmann, Rauch, Pfeiffer, & Doerr, 2004), and the discordant mosaicism rate in twins is associated with the duration of separation from the mother (Wachtel, Somkuti, & Schinfeld, 2000). To date, only six cases of monozygotic twins with 45,X/46,XY mosaicism and discordant for phenotypic sex, have been reported (Arizawa, Suehara, Takemura, & Nakayama, 1988; Costa et al., 1998; Gonsoulin, Copeland, Carpenter, Hughes, & Elder, 1990; Karp, Bryant, Tagatz, Giblett, & Fialkow, 1975; Reindollar, Byrd, Hahn, Haseltine, & McDonough, 1987). Because many of the patients are alive, mosaicism of the above patients was analyzed in specific cells, such as lymphocytes and fibroblasts. Therefore, there are few reports regarding the status of mosaicism in organs consisting of ectoderm, mesoderm, and endoderm, specifically in cases of 45,X/46,XY mosaicism.

We studied a case of monozygotic twins with 45,X/46,XY mosaicism and different external genitalia; we hypothesized that the differences in external genitalia were due to differences in the degree of 45,X/46,XY mosaicism in the organs. Here, we describe the clinical findings and cytogenetic and molecular analyses of this rare case of 45,X/46,XY mosaicism and discuss its underlying etiology.

## 2 CLINICAL REPORT

**Figure 1:**
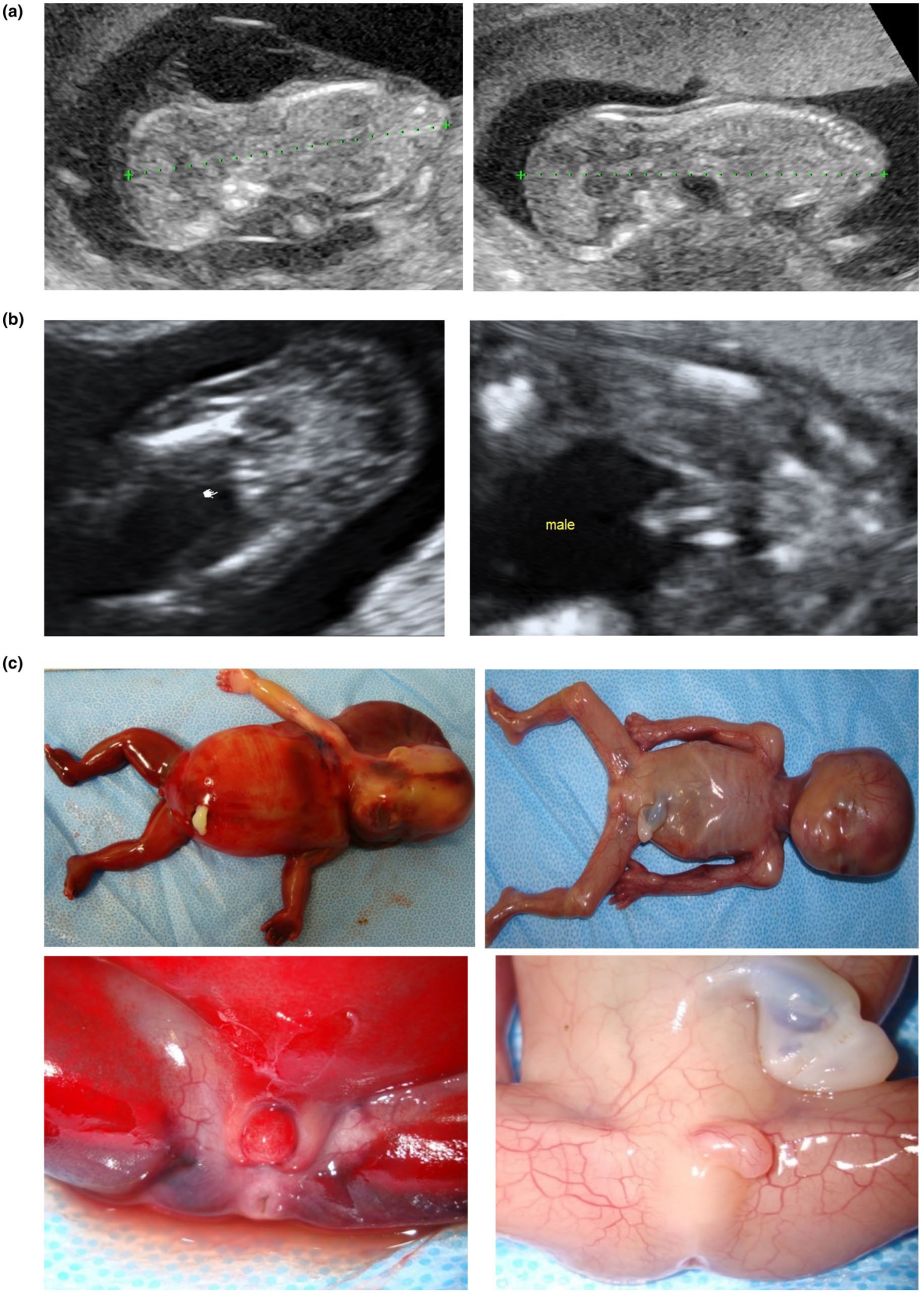
(a) Ultrasonography images revealing cystic hygroma in fetus 1 (left) and healthy fetus 2 (right). (b) Ultrasonography images of fetuses with different external genitalia. External genitalia of female fetus 1 (left) and male fetus 2 (right). (c) In fetus 1 (left), severe cystic hygroma and clitoral hypertrophy were detected. In fetus 2 (right), major anomalies could not be detected without bilateral cryptorchidism

A 37‐year‐old primigravida woman with no previous medical history was referred to the obstetrics department at the National Center for Child Health and Development at 12 0/7 weeks of gestation for fetal evaluation. She had been consuming folic acid and multivitamins before pregnancy and underwent intracytoplasmic sperm injection with a single embryo transfer. Ultrasonography revealed monochorionic diamniotic twins and severe cystic hygroma in one of the fetuses (Figure 1a). The patient had no family history of multiple pregnancies, fetal malformations, or miscarriage. After counseling with clinical geneticists, chorionic villus sampling was performed at 12 5/7 weeks of gestation, revealing a 45,X/46,XY karyotype. Since it has been reported that cystic hygroma on prenatal ultrasound is attributable to Turner syndrome in 30%–70% of cases (Alpman et al., 2009), at this point, the cystic hygroma was assumed to be caused by Turner mosaicism. However, it was not clear whether both fetuses were Turner mosaics, or if one fetus was Turner mosaic and the other was not. Moreover, we also demonstrated the possibility of confined placental mosaicism in the parents. At 17 2/7 weeks of gestation, ultrasonography detected that the fetus with cystic hygroma had external female genitalia while the other fetus had external male genitalia (Figure 1b). These findings suggested that the female fetus with cystic hygroma was Turner mosaic, whereas the male fetus was either normal or Turner mosaic with low mosaicism. During the genetic counseling sessions, we explained the impossibility of performing selective feticide of only one fetus in Japan and that the only choices were to either continue the pregnancy or abort both fetuses. Moreover, we proposed that the patient receive amniocentesis, but she denied the proposal. After repeated genetic counseling sessions, the patient and her husband opted for an abortion at 18 4/7 weeks of gestation. An autopsy revealed a normal penis and testes in one fetus and a normal vagina, uterus, and ovaries in the other fetus, which also had severe cystic hygroma (Figure 1c). No streak gonads were diagnosed pathologically in either fetus. The vascular anastomoses between the twins were confirmed by pathological examination of the placenta. This pathological finding suggested that the monochorionic diamniotic twins originated from monozygotic twins.

We examined the genetic background of the fetuses to better understand the etiology of their condition. The Research Ethics Committee at the National Center for Child Health and Development approved this genetic study (Approval number: 234, 2206). Written consent was obtained from the patient for publication of the case. First, to confirm that the origin of these fetuses was monozygotic and to exclude the possibility of confined placental mosaicism in the villi, we collected DNA from each umbilical cord to compare single nucleotide polymorphisms (SNPs) using SNP‐array analysis (HumanCytoSNP‐12 v2.1, Illumina Inc.^®^). After confirming that all 274,099 SNPs, except probes on chromosomes X and Y, were matched between both fetuses, the two fetuses were determined to be genetically monozygotic twins. Assessment of structural abnormalities in the fetal chromosomes revealed that only one loss of heterozygosity (LOH) was observed on chromosome 11, ranging from 47,976,882 to 51,530,241 bp. None of the genes in the LOH region was thought to play a role in the development of the genitalia and fetal anomaly. Second, to investigate the cause of the discordant sex phenotype between the fetuses and to confirm the diagnosis of Turner mosaicism, we counted the number of 46,XY and 45,X cells in the internal genitalia, liver, heart, lungs, adrenal glands, bone marrow, and spine, using fluorescence in situ hybridization with XY probes (XY‐FISH) (Vysis CEP X (DXZ1) SG/Y (DYZ3) SO Probes, Abbott Laboratories, Chicago, IL, USA). The two fetuses presented discordant mosaicism for 45,X and 46,XY (Table 1). There was a statistically significant difference in the distribution of mosaicism between the organs of the twins (Chi‐square test, *p* < .001). These results indicated that both fetuses were Turner mosaic, and the discordant sex phenotype was due to differences in the degree of mosaicism.

**Table 1:** Percentages and cell counts of the 45,X/46,XY karyotype by XY‐FISH in several organs

External genitalia	Karyotype	Sample material (% (Cell counts))							Total (% (Cell counts))
		Ectoderm	Ectoderm/Mesoderm	Mesoderm			Endoderm		
		Spine	Adrenal grand	Bone marrow	Heart	Gonad	Liver	Lung	
Fetus1 (female twin)	45,X	93.4 (113)	88.7 (47)	97.6 (80)	89.3 (75)	98.2 (165)	35.4 (121)	98.6 (485)	80.9 (1,086)
	46,XY	6.6 (8)	11.3 (6)	2.4 (2)	10.7 (9)	1.8 (3)	64.6 (221)	1.4 (7)	19.1 (256)
Fetus2 (male twin)	45,X	20.0 (29)	23.3 (41)	26.8 (33)	20.4 (32)	23.3 (68)	16.3 (76)	9.9 (39)	18.1 (318)
	46,XY	80.0 (116)	76.7 (135)	73.2 (90)	79.6 (125)	76.7 (224)	83.7 (389)	90.1 (356)	81.9 (1,435)

## 3 DISCUSSION

Monozygotic twins with 45,X/46,XY mosaicism, and different external genitalia, are described in the present report. We performed cytogenetic and molecular analyses and clarified the degree of mosaicism in the internal genitalia, liver, heart, lung, adrenal gland, bone marrow, and spine of the male and female fetuses. Results indicated that the abundance ratio of 45,X cells in male and female fetuses was approximately 20% and 80%, respectively. In our review of previous literature, we found reports of six monozygotic twins of different sex with 45,X/46,XY mosaicism (Table 2). Because four out of these six cases survived, only a few organ tissues, such as lymphocytes and fibroblasts, had been cytogenetically characterized. Unfortunately, because our case led to an outcome of induced abortion, several organ tissues—including the ectoderm, mesoderm, and endoderm—could be analyzed with more cell counts compared to other cases, and the degree of mosaicism could be more precisely revealed.

**Table 2:** Cytogenetic analysis data regarding previously published case reports and the presented case of monozygotic twins with 45,X/46,XY mosaicism and discordant sex phenotype

Case	Author	Year	Total mosaic rate						Gonad mosaic rate						Other analyzed organs	Time of karyotyping	Outcome
			Female twin			Male twin			Female twin			Male twin					
			45,X(%)	46,XY(%)	Cell counts(*n*)	45,X(%)	46,XY(%)	Cell counts(*n*)	45,X(%)	46,XY(%)	Cell counts(*n*)	45,X(%)	46,XY(%)	Cell counts(*n*)			
1	Karp et al	1975	63.3	36.7	30	2.0	98.0	50	100.0	0.0	19	NA	NA	NA	Fo (Cell counts not included)	Postnatal	Alive
2	Reindollar et al	1987	45.4	54.6	240	12.6	87.4	349	44.3	55.7	140	0.0	100.0	50	Fi, Ly	Postnatal	Alive
3	Arizawa et al	1988	NA	NA	NA	NA	NA	NA	NA	NA	NA	NA	NA	NA	Fi, Ly	Postnatal	Spontaneous Abortion
4	Gonsoulin et al	1990	49.1	50.9	110	12.0	88.0	100	NA	NA	NA	NA	NA	NA	Fi, Fb, K, Ly, Lu, P	Prenatal and Postnatal	Artificial Abortion
5	Costa et al. (case I)	1998	86.0	14.0	164	40.3	59.7	62	100.0	0.0	104	NA	NA	NA	Fi, Ly	Postnatal	Alive
6	Costa et al. (case II)	1998	39.9	60.1	173	5.1	94.9	79	100.0	0.0	25	0.0	100.0	25	Fi, Ly	Postnatal	Alive
7	Presented case	2020	80.9	19.1	1,342	18.1	81.9	1753	98.2	1.8	168	23.3	76.7	292	A, B, C (Cell counts not included), H, Li, Lu, S	Prenatal and Postnatal	Artificial Abortion

Note

Gonad includes ovary, testis, vas deferens, and streak gonads.

Abbreviations: A, adrenal grand; B, bone marrow; C, chorionic villus; Fb, fetal blood; Fi, fibroblasts; Fo, fallopian tube; H, heart; K, kidney; Li, liver; Lu, lung; Ly, lymphocytes; NA, not available; P, placenta; S, spine.

Cytogenetic studies of the female fetus showed a high percentage of 45,X cells in all organs except the liver (Table 1). In contrast, in the male fetus, the percentage of 46,XY cells was high in all organs. It is unclear why there was a difference in the degree of mosaicism only in the liver, though it is possible that blood cells were also counted. Hsu (1994) reported that a high percentage of 45,X cells in peripheral lymphocytes was correlated with the female phenotype. Although we did not analyze lymphocytes, our results revealed that the female fetus had a high percentage of 45,X cells in all organs except the liver. While our results complement those reported by Hsu (1994), a high percentage of total 45,X cells is not always related to the female phenotype (Wu et al., 2017). It is also unclear whether there is a difference in the incidence of 45,X/46,XY mosaicism in cells of singletons and twins. However, based on our results and past case reports, the high percentage of 45,X cells of the gonads may be related to the female phenotype (Table 2).

Since all SNP probes were matched in both fetuses, the monochorionic diamniotic twins were confirmed to be monozygotic twins. This suggested that one embryo with the 46,XY karyotype was split into 45,X and 46,XY cell lines. As both fetuses presented 45,X/46,XY mosaicism, an abnormal mosaic embryo was likely to be present before splitting. Imbalanced inner cell mass splitting was presumed to cause the abundance of mosaicism in the twins.

Owing to the rarity of monozygotic twins with different genitalia, only a few cases diagnosed prenatally have been reported. Usually, the presence of different genitalia in twin fetuses suggests dizygous twins. However, as in this case, monozygotic twins with different genitalia can exist. Therefore, obstetricians may consider complications, such as twin‐to‐twin transfusion syndrome and twin anemia‐polycythemia sequence, for such cases.

Despite the limited information available about the prenatal diagnosis of 45,X/46,XY mosaic twins, the parents in this case opted for artificial abortion after genetic counseling. According to previous literature, many cases of 45,X/46,XY mosaic twins are alive and live normally. The prenatal case of 45,X/46,XY monozygotic twins with different sex phenotypes is extremely rare, and pregnancy and postnatal course are not well known. These factors may have caused the parents anxiety and could have influenced their decision to select artificial abortion. Although the parents opted for an artificial abortion in this case, the etiology and laboratory findings obtained from this case provide valuable information for future genetic counseling.

## 4 CONCLUSIONS

In summary, we report an extremely rare case of monozygotic twins with a 45,X/46,XY karyotype and confirmed that this discordant sex phenotype can be observed in cases that do not involve dizygotic twins. Differences in the degree of mosaicism might be related to the female fetus exhibiting symptoms of Turner syndrome. Moreover, cases who have survived without problems have also been reported. It is, therefore, necessary to address pregnancy management after accurately communicating these facts to parents. Moreover, cytogenetic analyses of the case presented here provide new insight into the cause of this phenotype that can undoubtedly be used for genetic counseling.

## CONFLICTS OF INTEREST

The authors declare that they have no conflicts of interest.

## AUTHOR'S CONTRIBUTION

Contributors SW was responsible for the organization and coordination of the report. KT was the chief investigator and also responsible for the data analysis. KT, TS, MN, AS, KT, OM, SW, KH, and HS developed the study design. All authors contributed to the writing of the final manuscript. All members of this Study Team contributed to the management or administration of the report.

## Data Availability

The data sets generated during and/or analyzed during the current study are not publicly available due to personal information but are available from the corresponding author on reasonable request.
